# Review and prospect of the biomedical effects of hydrogen

**DOI:** 10.1186/s13618-014-0019-6

**Published:** 2014-11-29

**Authors:** Xiao Zhai, Xiao Chen, Shigeo Ohta, Xuejun Sun

**Affiliations:** Graduate Management Unit, Changhai hospital affiliated to the Second Military Medical University, Shanghai, PR China; Department of Orthopedics, Changhai hospital affiliated to the Second Military Medical University, Shanghai, PR China; Department of Biochemistry and Cell Biology, Institute of Development and Aging Sciences, Graduate School of Medicine, Nippon Medical School, 1-396 Kosugi-machi, Nakahara-ku, Kawasaki-city, Kanagawa-ken 211-8533 Japan; Department of Navy Aeromedicine, Second Military Medical University, 168 Changhai, Shanghai, China

**Keywords:** Hydrogen, Antioxidant, Reactive oxygen species, Inflammatory, Apoptosis, Necrosis, Physiological effect

## Abstract

Hydrogen, the simplest gas in nature, was recently reported as a therapeutic antioxidant through selectively reducing cytotoxic oxygen radicals. Though hundreds of studies on curative effects of hydrogen were published and justified, the mechanism remains unclear. We proposed several promising directions in this area by relatively in-depth analysis. Firstly, the physiological function of hydrogen was regarded neutralizing free radicals at a low dose; however, physiological effects of an excessive dose of hydrogen were necessary for the comprehensive understanding. Secondly, the therapeutic effects and mechanisms were explained by anti-oxidative, anti-inflammatory and apoptosis ways, while the limitation was obvious and needed update. Thirdly, further studies might be focused on the possible networks including effecters and receptors of hydrogen, and the evolutionary perspective was a good point of view. In conclusion, this review might be a reference and guidance for relative scholars.

## Introduction

Hydrogen(H_2_) is the simplest and widely distributed element in nature, and the most abundant ingredient of human body composition. Hydrogen gas is colorless, odorless and tasteless, and was believed physiologically inert in the body before. However, in 2007 Oshawa *et al.* from Japan reported that hydrogen could ameliorate cerebral ischemia-reperfusion injury and selectively reduce cytotoxic oxygen radicals including hydroxyl radical (•OH) and peroxynitrite (ONOO−) on Nature Medicine [1], which provoked a worldwide intensive attention and overturned our previous cognition. It was then hypothesized that hydrogen might be not only a resource for clean energy but also a promising therapeutic medicine. Till now, more than 400 papers and reviews have already been published in this field on dozens of diseases, involving various types of organ and tissue ischemia, atherosclerosis, senile dementia, neural degenerative diseases and so on [2-4].

However, a few problems were encountered for further investigations. Firstly, the probable signal transduction pathways were indecisive. It was still wondered whether hydrogen acted with free radicals directly or initiated the effectors to ameliorate the injuries. Secondly, the experiments before were mainly practiced on animals, while clinical verification and randomized controlled double-blinded researches on human beings were still uncertain.

Therefore, it was high time that we make breakthroughs to give the biological study on hydrogen an extra boost. Since the underlying mechanisms were very important to help deepen the understanding, guide the future study, confirm more candidate disease models and extend the application, and as the study group having published the most related essays, we raised some promising research models and directions as a reference for scholars in this field.

### Physiological function of hydrogen

Oxidative injuries occur with a significant increase of various kinds of reactive oxygen species(ROS) [5], including hydrogen dioxide (H_2_O_2_), superoxide anion (O_2_−), ·OH, nitric oxide (NO•), and ONOO−. The ROS breaks the homeostasis of the oxidative and anti-oxidative arrangement and irretrievably drives downstream signaling networks, leading to peroxidation of nucleic acids or fatty acids, removal of protein cross-linking, and apoptosis of cells finally.

H_2_ is non-inflammable nor non-explosive when its concentration is below 4.1% in pure oxygen or 4.6% in air [1]. In 2007 Ohsawa *et al.* showed that H_2_ was a unique free radical scavenger, which selectively reduced •OH and ONOO−, maintaining the physiological homeostasis in cells such as reducing destruction of various anti-oxidases and deoxyribonucleic acid(DNA) and ensuring the normal function of signalization. Animal studies showed that inhalation of 2% hydrogen or intraperitoneal injection of saturated hydrogen saline could selectively scavenge ROS, inhibit inflammation and reduce oxidative damage [6,7].

However, in-vitro evidences of selective anti-oxidative effects still required direct conviction and sufficiency, for the most unequivocal evidence before was acquired in solution without repeated experiments [8-10]. Meanwhile, the in-vivo studies at present, which observed the changes of effects or molecules, were also the downstream action of ROS [11].

On the other hand, it is also important to identify the physiologically side effects of hydrogen, in view of the fact that we should consider a widely accepted biological law, which is as long as the dose exceeds a certain limitation, it reverses its course. It seems unreasonable that hydrogen treats diseases without any physiological side effects. Meanwhile, the studies on the side effects would help us to understand the biological function of hydrogen more comprehensive.

### Therapeutic effects and mechanisms of hydrogen

Hydrogen was supposed to ameliorate dozens of diseases. Except the property of selectively reducing oxygen radicals, its effects of anti-inflammation [2,12,13] and anti-apoptosis [14,15] were also regarded importantly. Oxidative stress is relevant to inflammation and apoptosis due to free radicals would impair cells and produce many inflammatory factors, which acts as an important starting aspect in cell death. The downstream cascade reaction of the therapeutic effects might reveal the deep protective mechanisms and net works of hydrogen.

For anti-oxidative ways, anti-oxidases were considered participating positively. Apart from neutralization with free radicals directly, it was reported that hydrogen increased the expression of anti-oxidases both in in-vivo [16] and in-vitro [17]. Nuclear factor - erythroid 2-related factor 2(Nrf2), the key transcription factor like the commander-in-chief in endogenous anti-oxidative system, was validated a hot spot and an important effecter [18]. Once the Nrf2 is activated after the administration of hydrogen, it initiates the endogenous anti-oxidative system, including expressions of uperoxide dismutase(SOD) [16] and glutathione(GSH) [19]. For example, heme oxygenase-1(HO-1), as one of critical downstream molecule of Nrf2, was found to regulate oxidative stress in a range of diseases, such as neurodegenerative disease [20] and in LPS-stimulated RAW 264.7 macrophages [17].

On the other hand, the endogenous anti-oxidative system was different from the exogenous anti-oxidative drugs or exogenous hydrogen. Generally, exogenous drugs cannot activate endogenous anti-oxidase, for the reason that in a feedback manner, exogenous anti-oxidants might ameliorating ROS while inhibit endogenous anti-oxidative system since ROS are significant major factors in activating endogenous anti-oxidative system. So, it should be determined whether ROS or the endogenous anti-oxidative hydrogen weights more in the protective role. Based on the discussion above, it might be hypothesized that hydrogen mobilizes the endogenous anti-oxidative system through activating Nrf2 expression besides playing the direct anti-oxidative role with ROS.

For anti-inflammatory effects, hydrogen was proved suppressing various inflammatory cytokines. Du Z *et al.* [21] showed that hydrogen-rich saline could reduce the level of interleukin(IL)-6, tumor necrosis factor-α(TNF-α) and malondialdehyde(MDA) in plasma. Yoon *et al.* [22] reported that the levels of Th2 cytokine, IL-5, and proinflammatory cytokines such as TNF-α and IL-6 in Hydrogen-fed mice were significantly lower than in control and Purified Water-fed mice. Buchholz *et al.* [2] reported that H_2_ can downregulate the expressions of pro-inflammatory cytokines such as TNF-α, IL-6, IL-1β, chemokine(C-C motif) ligand 2(CCL2). Liu *et al.* [23] proved that the pro-inflammatory cytokines (TNF-α, IL-1β and high mobility group box-1 protein (HMGB1)), anti-inflammatory cytokine (IL-10) were regulated by hydrogen. However, the networks of the effects are still unclear, and to find the receptors or effecters is the task in the future.

Effects of apoptosis are less predominant since the result of cell death is concentrated from various extremely harmful factors. It was hypothesized that accumulation of ROS is one of the several means to cause the apoptotic process, since the increasing of ROS releases arachidonic acids and provokes lipid peroxidation, causing damages to cells. Standing on this point of view, H_2_ can suspend neuronal apoptosis by neutralizing ROS, which has been investigated in many studies. Cai *et al.* demonstrated that H_2_ presented the ability of neuroprotection by reducing HI-induced caspase-dependent apoptosis in *in vivo* [15]. And in the following study, they found that the lipid peroxidation level was reduced, and neural apoptosis was thus decreased due to H_2_ administration [7].

### Future Mechanism studies for hydrogen

The therapeutic effects of hydrogen were observed and verified. However, the molecule mechanism was uncertain and challengeable. It is important to discuss how to design and perform the specific experiments in the future.

We define the direct effects of hydrogen by neutralizing ROS and the indirect effects by activating possible proteins. Further studies on indirect effects should take the priority.

Firstly, the existence of hydrogen is transient in the body. Hydrogen would be exhaled and removed in about 30 min as long as the administration is stopped [24]. Many results showed that a short time exposure of hydrogen had durative and long term effects [25,26]. The long term therapeutic effects could not be explained with such a short time of administration, so it indicated that hydrogen possessed indirect effects instead of reducing ROS. To prove this, preconditioning studies are recommended. Preconditioning studies were mostly used in observing indirect effects of one particular factor. In this experiment, animals and cells should be first preconditioned by hydrogen for several days and then received the injury operation. Once the protective effects of preconditioning hydrogen are proved, it can be identified that hydrogen has indirect effects rather than directly decreasing ROS. The study might overthrow the selectively anti-oxidative theory and raise multiple mechanisms more than only neutralizing toxic ROS.

Secondly, it was also unidentified physiologically whether hydrogen can activate molecules such as Nrf2, ghrelin, FGF21, HO-1 and so on in healthy animals and human. If physiologically not, it might indicate that the molecules are induced by oxidative stress rather than effecters of hydrogen. To confirm this, using specific blockers and gene knocking-out techniques are ideal methods: reduced effects will prove that hydrogen physiologically activate these molecules.

Thirdly, effecter factors that hydrogen activates should be investigated, which is more difficult. Methods of omics might be used to seek interesting molecule. Luckily, various oxido-reductase have been found participating in the effects of hydrogen.

Furthermore, to investigate the mechanism of hydrogen, evolutionary perspective is a creative and good point. First, most of the bacteria and lower animals and plants can produce and utilize hydrogen, so it is feasible that hydrogen might be biologically active in human. Second, bacterial enzymes producing hydrogen are the current highlight in the hydrogen energy field, and molecule details in the hydrogen producing are very clear [27,28]. Last but not the least, during the evolutionary process, these molecules or enzymes might have residual sites for hydrogen.

## Conclusion

It is leading to the guess that hydrogen is a substance for physiological accommodation as a novel antioxidant gas with medical applications, following NO, CO, and H_2_S. The physiological function of a small amount of hydrogen was regarded neutralizing free radicals; however, an excessive dose of hydrogen might be investigated for comprehensive physiological effects. To explain for the therapeutic effects and mechanisms, antioxidant, anti-inflammatory and apoptosis ways were introduced while the limitation was obvious and needed update. In addition, further studies might be focused on the possible networks including effecters and receptors of hydrogen, and the evolutionary perspective is a good point of view. In short, the hydrogen therapy might be an alternative in the clinic. The field of hydrogen biology has a clear objective, a bright future and arduous tasks and our hard work in this area is a decisive contribution for human health.
